# Turning pancreatic cancer from cold to hot: the promise of a p53-expressing oncolytic adenovirus (OBP-702)

**DOI:** 10.1007/s10147-026-02991-7

**Published:** 2026-02-21

**Authors:** Shinji Kuroda, Hiroshi Tazawa, Masashi Hashimoto, Nobuhiko Kanaya, Yoshihiko Kakiuchi, Shunsuke Kagawa, Yasuo Urata, Toshiyoshi Fujiwara

**Affiliations:** 1 Department of Gastroenterological Surgery, Okayama University Graduate School of Medicine, Dentistry and Pharmaceutical Sciences, 2-5-1 Shikata-Cho, Kita-Ku, Okayama, 700-8558 Japan; 2 Center for Innovative Clinical Medicine, Okayama University Hospital, Okayama, Japan; 3 Oncolys BioPharma Inc, Tokyo, Japan

**Keywords:** Oncolytic adenovirus, p53, OBP-702, Immunogenic cell death, Tumor microenvironment, Pancreatic cancer

## Abstract

Pancreatic cancer remains one of the most lethal malignancies, with limited therapeutic options and poor responsiveness to immune checkpoint inhibitors (ICIs). This resistance is largely attributed to its profoundly immunosuppressive and desmoplastic tumor microenvironment (TME), characterized by low tumor mutational burden, dense stroma, and abundant immunosuppressive cell populations. Therefore, strategies capable of enhancing tumor immunogenicity and overcoming immune evasion are urgently needed. Oncolytic virotherapy is a promising approach, offering not only tumor-selective cytotoxicity, but also potent immunomodulatory effects. Of these agents, Telomelysin (OBP-301, Suratadenoturev), a telomerase-specific oncolytic adenovirus, demonstrated clinical safety but limited efficacy in refractory tumors. To address this challenge, we developed OBP-702, a next-generation, p53-armed, oncolytic adenovirus designed to augment antitumor activity. Preclinical studies have shown that OBP-702 exerts robust cytotoxicity through multiple mechanisms, including p53-mediated apoptosis and autophagy, E1A–E2F1-mediated p21 suppression, and inhibition of oncogenic KRAS pathways. Importantly, OBP-702 induces strong immunogenic cell death, activates dendritic cells, and promotes tumor-specific T-cell responses, effectively converting immunologically “cold” pancreatic tumors into “hot” tumors. OBP-702 also remodels the immunosuppressive TME by reducing granulocyte–macrophage colony-stimulating factor (GM-CSF) secretion, suppressing myeloid-derived suppressor cells (MDSCs), and targeting stromal components, such as cancer-associated fibroblasts (CAFs). These effects contribute to enhanced responses to ICIs and standard chemotherapies. Given its multifaceted antitumor functions and ability to overcome key barriers in pancreatic cancer, OBP-702 represents a highly promising therapeutic candidate. A first-in-human clinical trial evaluating endoscopic ultrasonography-guided intratumoral injection of OBP-702 is currently in preparation, expected to advance clinical translation of this novel virotherapeutic strategy.

## Introduction

Pancreatic cancer is a highly lethal malignancy with a 5 year overall survival rate of approximately 10% [1]. Although it ranks as the twelfth most common cancer in terms of incidence, it is the sixth leading cause of cancer-related death worldwide [2]. Pancreatic cancer is often diagnosed at an advanced stage, at which point it is frequently deemed unresectable. Although advancements in chemotherapy have led to some improvement in survival, the overall prognosis remains poor and far from satisfactory. Recently, immune checkpoint inhibitors (ICIs) targeting checkpoint molecules such as cytotoxic T-lymphocyte antigen-4 (CTLA-4) and programmed cell death 1 (PD-1)/programmed death-ligand 1 (PD-L1) have been shown to significantly improve survival in certain cancer types, including melanoma and lung cancer [3]. However, clinical trials have reported that pancreatic cancer is resistant to ICI-based immunotherapy [4]. Therefore, it is crucial to understand the tumor microenvironment (TME) associated with the immune resistance of pancreatic cancer, which is considered an immunologically “cold” tumor, and to develop therapeutic strategies that stimulate immune-activating factors and remodel the immunosuppressive TME to improve patient outcomes.

Oncolytic virotherapy, which utilizes genetically engineered viruses as anticancer agents by conferring tumor selectivity to their replicative capacity, has attracted increasing attention. In recent years, strategies involving the incorporation of therapeutic genes into oncolytic viruses to achieve multifaceted antitumor effects have been actively pursued. H101 (Oncorine), a recombinant adenovirus with a deletion of the E1B-55 kDa gene and a portion of its E3 region, designed to selectively replicate in and lyse tumor cells with p53 deficiency or mutation, became the first oncolytic virus approved in China in 2005 for the treatment of nasopharyngeal carcinoma [5]. Talimogene laherparepvec (T-VEC and IMLYGIC), a herpes simplex virus (HSV) that carries the gene for granulocyte–macrophage colony-stimulating factor (GM-CSF) to stimulate immune activation, demonstrated good outcomes in clinical trials for malignant melanoma and became the first oncolytic virus to gain regulatory approval in both the United States and Europe in 2015 [6–8]. In Japan, teserpaturev (G47Δ, Delytact), a genetically engineered HSV, has received conditional approval for the treatment of glioblastoma [9].

**Fig. 1:**
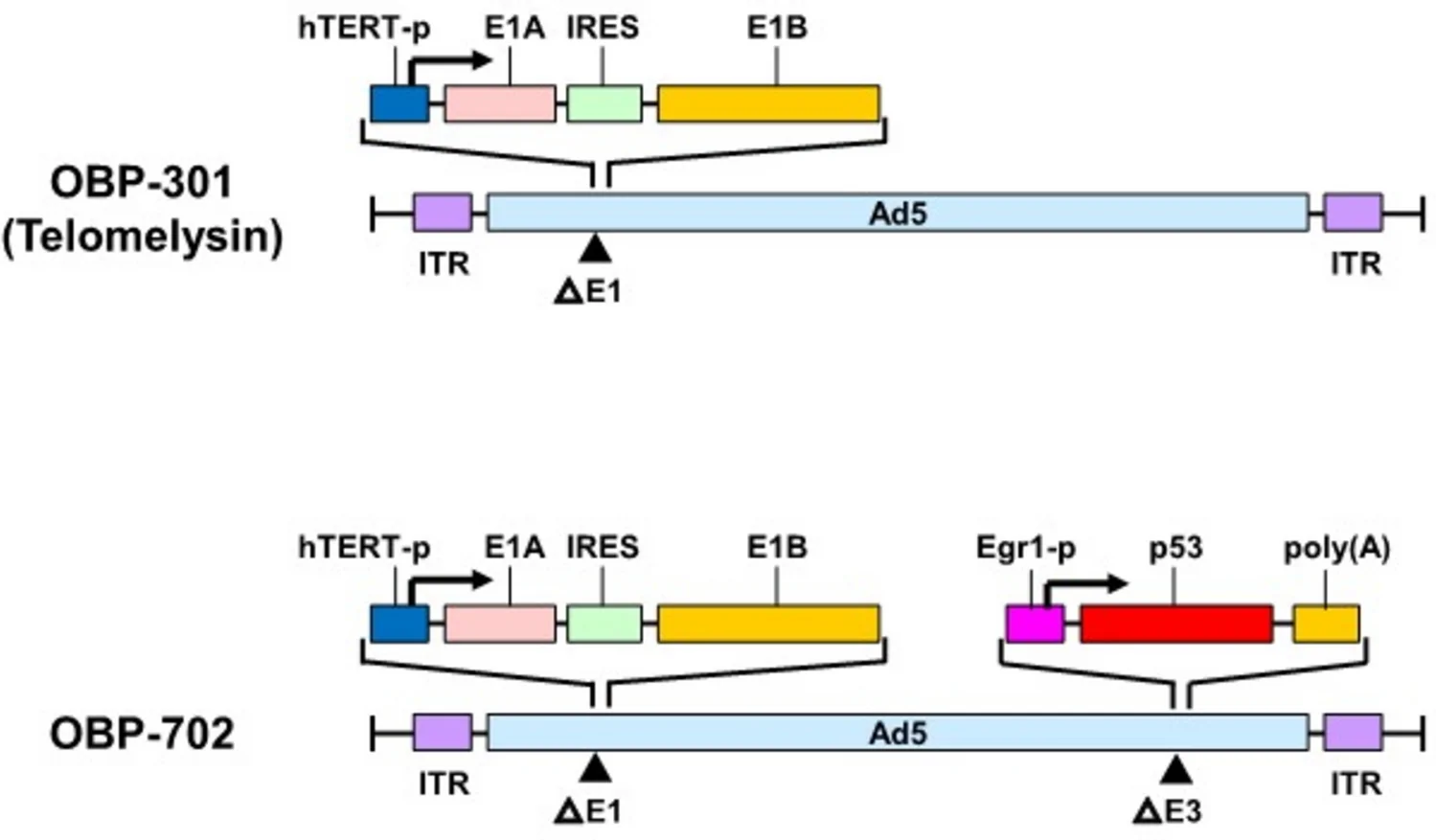
Gene structures of OBP-301 and OBP-702

Telomelysin (OBP-301, Suratadenoturev) (Fig. 1), a telomerase-specific oncolytic adenovirus that we developed, is designed to selectively replicate in cancer cells and induce tumor lysis under the control of the human telomerase reverse transcriptase (hTERT) promoter, which is highly active in cancer cells [10–13]. The safety of Telomelysin was demonstrated in a phase I clinical trial for various solid tumors conducted in the United States since 2006, under the approval of the U.S. Food and Drug Administration (FDA) [14]. Following a preclinical study demonstrating the synergistic effects of Telomelysin and radiation therapy against esophageal cancer [15, 16], and a phase I clinical trial confirming the safety of this combination [17], a phase II clinical trial evaluating the combination in patients with advanced esophageal cancer demonstrated its efficacy. Based on this evidence, a regulatory approval application is currently under review, and the broad clinical implementation of viro-radiotherapy (VRT), which combines virotherapy and radiotherapy, is highly anticipated in the future. Although Telomelysin is a minimally invasive and effective biologic agent, its therapeutic efficacy has been found to be limited in treatment-refractory cancers, such as pancreatic cancer. Based on this background, and focusing on the diverse functions of the p53 tumor suppressor gene [18], we developed OBP-702 (Fig. 1), a modified adenoviral agent engineered by incorporating the p53 gene into Telomelysin [19], and we have since been investigating its therapeutic efficacy against treatment-refractory cancers, including pancreatic cancer.

This review introduces the therapeutic efficacy and molecular mechanisms of OBP-702 in the treatment of pancreatic cancer, with a particular focus on its ability to enhance antitumor immunity and modulate the immunosuppressive TME. Finally, we provide an overview of the current clinical development of oncolytic viruses for pancreatic cancer and outline future perspectives for OBP-702.

### Cytotoxic potential through diverse molecular mechanisms by OBP-702

OBP-702 is a telomerase-specific oncolytic adenovirus engineered to express the p53 tumor suppressor gene, which is frequently mutated or inactivated in a wide range of human cancers, including pancreatic cancer [19]. It replicates selectively in cancer cells with elevated telomerase activity while sparing normal cells that lack such activity. This telomerase-dependent replication enables OBP-702 to serve as a tumor-selective vector, minimizing off-target toxicity and maximizing its therapeutic index. The cytotoxic effects of OBP-702 are mediated primarily through p53-dependent pathways. On infection, OBP-702 induces p53 expression in cancer cells, which in turn activates downstream targets, such as BAX and DRAM, leading to the induction of apoptosis and autophagy [20]. In addition to its p53-mediated cytotoxic effects, OBP-702 exerts broader molecular effects through viral components. Accumulation of the early viral protein E1A activates the transcription factor E2F1, which promotes the expression of microRNAs, such as miR-93 and miR-106b. These microRNAs suppress p21, a cyclin-dependent kinase inhibitor, thereby removing a key checkpoint in the cell cycle and further amplifying the apoptotic and autophagic cell death processes [20]. Importantly, OBP-702 has shown efficacy not only in p53-mutant tumors through forced expression of functional p53, but also in tumors harboring intact but suppressed p53 pathways, where reactivation can restore tumor suppressive functions.

Another characteristic genetic alteration in pancreatic cancer is the high frequency of activating mutations in the oncogene KRAS, observed in over 90% of cases [21]. These mutations result in constitutive activation of downstream signaling cascades, such as the RAF–MEK–ERK and PI3K–AKT pathways. Persistent activation of these pathways promotes uncontrolled cell proliferation, survival, migration, and invasion. Furthermore, KRAS mutations are associated with resistance to many conventional chemotherapeutic agents and are widely regarded as among the key drivers of pancreatic tumorigenesis and therapeutic resistance. Accordingly, clinical trials of KRAS inhibitors targeting KRAS mutations in pancreatic cancer are currently underway [22]. OBP-702 has been shown to robustly suppress KRAS–ERK–MEK signaling in human pancreatic cancer cell lines harboring various KRAS mutations [23]. This inhibition results in a marked reduction in cellular proliferation, motility, and invasive potential. Notably, the suppression of oncogenic signaling appears to be mediated by p53 restoration, since p53 has been reported to antagonize RAS signaling through multiple mechanisms, including the transcriptional activation of MAPK pathway inhibitors and the repression of downstream effectors. In this context, OBP-702 may exert its effects by restoring tumor suppressor networks that counterbalance the oncogenic drive of mutant KRAS. This highlights the therapeutic versatility of OBP-702 across various genetic backgrounds and underscores its potential as a novel and effective treatment modality for pancreatic cancer.

### Activation of antitumor immunity and long-term immunity by OBP-702

Pancreatic cancer is widely recognized as an immunologically “cold” tumor, characterized by low immunogenicity and poor responsiveness to ICIs [24]. This immunoevasive phenotype is largely attributed to the tumor’s low tumor mutational burden (TMB), which limits the generation of neoantigens capable of eliciting effective T-cell responses. In this context, strategies to enhance the immunogenicity of pancreatic cancer cells are urgently needed. One promising approach is the induction of immunogenic cell death (ICD), a regulated form of cell death that stimulates an adaptive immune response against tumor antigens. ICD is characterized by the release of damage-associated molecular patterns (DAMPs), such as ATP, HMGB1, and calreticulin, which serve as immunostimulatory signals to recruit and activate antigen-presenting cells, thereby initiating the cancer immunity cycle [25].

We previously demonstrated that Telomelysin and its fiber-modified derivative, OBP-502, can strongly induce ICD in murine pancreatic cancer cells [26]. This was shown by the release of ATP and HMGB1 following viral infection, indicating the activation of innate immune pathways. Importantly, OBP-702 was found to induce ICD even more robustly in both human and murine pancreatic cancer cells, which is attributed to p53-mediated mechanisms [27]. It also activates dendritic cells via tumor-derived extracellular vesicles following OBP-702 treatment, leading to the recruitment of cytotoxic T cells [28]. Furthermore, local administration of OBP-702 into the primary tumor was shown to trigger systemic antitumor immune activation, resulting in abscopal effects at distant, untreated metastatic sites. This phenomenon suggests that OBP-702 not only exerts direct cytolytic activity against infected tumor cells but also functions as an in situ cancer vaccine, converting immunologically “cold” tumors into “hot” ones through ICD-mediated antigen release and immune priming. Consistent with this mechanism, combination therapy with OBP-702 and ICIs further enhanced antitumor efficacy against pancreatic cancer, indicating a synergistic effect likely mediated by increased tumor immunogenicity and enhanced T-cell activation [27].

One of the key indicators of long-term antitumor immunity is the generation of memory T cells. Of the effector T cells responding to tumor antigens, a subset known as memory precursor effector cells (MPECs) gives rise to long-lived memory T cells, including central memory T cells (TCM), effector memory T cells (TEM), and tissue-resident memory T cells (TRM) [29]. Notably, CD8-positive, CD103-positive TRM cells have been associated with a good prognosis and are considered predictive biomarkers for the efficacy of immune checkpoint inhibitors [30, 31]. OBP-702 demonstrated a significant tumor growth-suppressive effect in a study evaluating its long-term immunological efficacy against murine pancreatic cancer models [32]. This antitumor effect was accompanied by a marked increase in antigen-responsive MPECs in the spleen and robust accumulation of CD8-positive TRM cells within the TME, strongly supporting the induction of localized, long-lasting immune memory. These TRM cells are thought to play a central role in maintaining immune surveillance and mediating rapid cytotoxic responses on tumor recurrence or progression. Importantly, this effect was also observed when OBP-702 was administered in combination with gemcitabine (GEM) and nab-paclitaxel (nab-PTX), one of the standard treatment regimens for pancreatic cancer. These findings suggest that OBP-702 may induce a durable antitumor immune response, offering long-term protection against tumor relapse in pancreatic cancer. Moreover, its ability to generate sustained immune activation may also support its potential use as a neoadjuvant therapy to improve surgical outcomes and prevent postoperative recurrence.

### Remodeling immunosuppressive TME by OBP-702

A hallmark of the TME in pancreatic cancer is the extensive proliferation of stromal tissue, predominantly composed of fibroblasts, along with poor angiogenesis that leads to hypoxia [33]. These conditions contribute to reduced infiltration and impaired function of cytotoxic T lymphocytes (CTLs) while simultaneously promoting the recruitment and activation of immunosuppressive cell populations, such as myeloid-derived suppressor cells (MDSCs) and regulatory T cells (Tregs) [24]. Furthermore, the intrinsically low tumor mutation burden (TMB) and downregulation of major histocompatibility complex (MHC) molecules reduce the immunogenicity of pancreatic cancer, allowing tumors to evade immune surveillance [34]. Consequently, the TME in pancreatic cancer becomes dominated by immunosuppressive elements rather than effector immune cells, creating a hostile environment for effective antitumor immunity and contributing to resistance to immunotherapy. Overcoming this immunosuppressive landscape is, therefore, a critical objective in improving the efficacy of immunotherapeutic strategies for pancreatic cancer.

We observed that human and murine pancreatic cancer cells with acquired resistance to GEM exhibited upregulated PD-L1 expression and promoted the accumulation of PD-L1^+^ MDSCs within the TME [35]. These findings suggest that GEM resistance not only diminishes the effectiveness of chemotherapy, but also reinforces immunosuppression within the tumor. Notably, intratumoral administration of OBP-702 into GEM-resistant murine pancreatic tumors significantly reduced the infiltration of MDSCs [35]. Mechanistic studies showed that OBP-702 downregulates the secretion of granulocyte–macrophage colony-stimulating factor (GM-CSF) by tumor cells, a key factor involved in the recruitment and expansion of MDSCs. In addition, OBP-702 enhanced the therapeutic efficacy of anti-PD-L1 antibody treatment in this resistant tumor model, indicating its potential as a combination partner to improve the response to immune checkpoint blockade. Together, these results suggest that OBP-702 can effectively remodel the immunosuppressive TME and help restore antitumor immune responses in pancreatic cancer, even in the setting of chemotherapy resistance.

OBP-702 has been shown to exert selective cytotoxic effects against cancer-associated fibroblast (CAF)-like pancreatic stellate cells, which contribute to stromal formation, through robust induction of p53 expression [36]. In an orthotopic, stroma-rich xenograft model using human pancreatic cancer cells, intratumoral administration of OBP-702 demonstrated pronounced antitumor activity, whereas the effect of Telomelysin was limited [23]. These findings suggest that OBP-702 selectively targets the tumor stroma within the pancreatic cancer microenvironment. By disrupting the abundant stromal components characteristic of pancreatic tumors, OBP-702 is also expected to enhance drug delivery efficiency within the tumor tissue.

Collectively, these findings highlight the multifaceted ability of OBP-702 to remodel the pancreatic TME by simultaneously suppressing immunosuppressive cell populations and targeting stromal components. Through these mechanisms, OBP-702 not only restores immune responsiveness, but also creates a more favorable context for the delivery and efficacy of combined therapies. Thus, OBP-702 represents a promising strategy to overcome the inherent therapeutic resistance associated with the desmoplastic and immunosuppressive nature of pancreatic cancer.

### Oncolytic viruses under clinical investigation for pancreatic cancer

Table 1 summarizes oncolytic viruses that have been or are currently being evaluated for their safety and efficacy in clinical trials, although none has yet reached clinical use for pancreatic cancer. Both T-VEC and H101, which have already been approved for clinical use in other cancer types, as mentioned earlier, have only completed phase I clinical trials in pancreatic cancer [37]. Canerpaturev (C-REV and HF10) [38], a naturally attenuated mutant strain of HSV-1 developed in Japan, has been evaluated in phase I clinical trials in combination with GEM plus erlotinib [39] or GEM plus nab-PTX. Pelareorep (Reolysin) [40], an oncolytic reovirus being developed as a systemically administered immuno-oncological viral agent, has been evaluated in several phase Ib and II clinical trials in combination with pembrolizumab, paclitaxel plus carboplatin, or gemcitabine [41–43]. Intravenous and intratumoral administration of ParvOryx (H-1PV)[44], an oncolytic H-1 parvovirus, has also been evaluated in a phase II clinical trial for metastatic pancreatic cancer [45]. In addition, hV01[46], a recombinant IL-21-expressing oncolytic vaccinia virus, is currently undergoing evaluation in a phase II clinical trial for advanced pancreatic cancer. Zabilugene almadenorepvec (VCN-01) [47], an oncolytic adenovirus engineered to replicate in tumors with a defective RB pathway, which features enhanced infectivity through a modified fiber and improved tumor distribution via the expression of a soluble hyaluronidase, has been tested in a randomized phase II clinical trial comparing GEM plus nab-PTX with or without VCN-01 for pancreatic cancer, following the confirmation of the safety and efficacy of this combination in a phase I clinical trial [48]. VCN-01 has been also tested in a phase I clinical trial in combination with huCART-meso, human chimeric antigen receptor modified T cells targeting mesothelin. Delolimogene mupadenorepvec (LOAd703) [49], an oncolytic adenovirus encoding two potent immunostimulatory transgenes of TMZ-CD40L and 4-1BBL, has been evaluated in a phase I/II clinical trial, with the phase I part testing its combination with gemcitabine plus nab-paclitaxel [50], and the phase II part assessing its combination with gemcitabine, nab-paclitaxel, and atezolizumab.

**Table 1:** Oncolytic viruses under clinical investigation for pancreatic cancer

Virus type	Name	Phase	Registration ID	Combination	Status	Reference
Herpes simplex virus	Talimogene laherparepvec (T-VEC, Imlygic)	I	NCT03086642		Completed	[37]
		I	NCT00402025		Completed	
	Canerpaturev (C-REV, HF10)	I	UMIN000010150	GEM + ERL	Completed	[39]
		I	NCT03252808	GEM + Nab-PTX	Active, not recruiting	
	OH2	I/II	NCT04637698		Terminated	
Reovirus	Pelareorep (Reolysin)	Ib	NCT02620423	Pembro + 5-FU/GEM/IRI	Completed	[41]
		II	NCT03723915	Pembro	Terminated	
		II	NCT01280058	PTX + CBDCA	Completed	[42]
		II	NCT00998322	GEM	Completed	[43]
Parvovirus	ParvOryx (H-1PV)	II	NCT02653313	iv and it	Completed	[45]
Vaccinia virus	hV01	II	NCT07006077		Recruiting	
Adenovirus	Zabilugene almadenorepvec (VCN-01)	I	NCT02045589	GEM + Nab-PTX	Completed	[48]
		I	NCT05057715	huCART-meso cells	Active, not recruiting	
		II	NCT05673811	GEM + nab-PTX	Completed	
	Delolimogene mupadenorepvec (LOAd703)	I/II	NCT02705196	II: GEM + nab-PTX II: GEM + nab-PTX + Atezo	Active, not recruiting	[50]
	H101 (Oncorine)	I	NCT05303090	Tislelizumab + Lenvatinib	Completed	

*GEM* gemcitabine, *ERL* erlotinib, *Nab-PTX* nab-paclitaxel, *Pembro* pembrolizumab, *5-FU* fluorouracil, *IRI* irinotecan, *PTX* paclitaxel, *CBDCA* carboplatin, *iv* intravenous administration, *it* intratumoral administration, *Atezo* atezolizumab

### Future perspectives for OBP-702

OBP-702 not only exerts cytotoxic activity through diverse mechanisms mediated by p53 expression, but it also strongly induces ICD and remodels the immunosuppressive TME, constructed by components, such as CAFs and MDSCs, into an immunologically “hot” environment. These multifaceted antitumor effects position OBP-702 as a highly promising therapeutic agent, particularly for treatment-refractory malignancies, such as pancreatic cancer, which are notoriously resistant to conventional and immune-based therapies. Based on these promising preclinical findings, a first-in-human, phase I clinical trial is being planned to assess the safety of endoscopic ultrasonography (EUS)-guided intratumoral injection of OBP-702 in patients with advanced pancreatic cancer refractory to standard chemotherapy. Despite the absence of notable adverse findings in preclinical studies, potential safety concerns remain in the clinical setting, including toxicity associated with enforced p53 expression and allergic reactions due to excessive immune activation. Accordingly, while the primary objective of this phase I clinical trial is to determine the maximum tolerated dose (MTD) based on dose-limiting toxicities, key safety considerations include whether the major adverse events observed with the predecessor agent Telomelysin, such as fever, fatigue, and lymphopenia, also occur with the p53-armed OBP-702, whether any novel adverse events associated with p53 expression emerge, and whether OBP-702 can be administered at the same dose level as the MTD established for Telomelysin. In parallel, exploratory analyses will be conducted to assess preliminary therapeutic efficacy and to identify predictive biomarkers that may inform patient selection and treatment monitoring in subsequent clinical stages. Although this phase I clinical trial will evaluate OBP-702 as monotherapy, it holds promise for future combination strategies with existing standard chemotherapies and ICIs.

## Conclusions

**Fig. 2:**
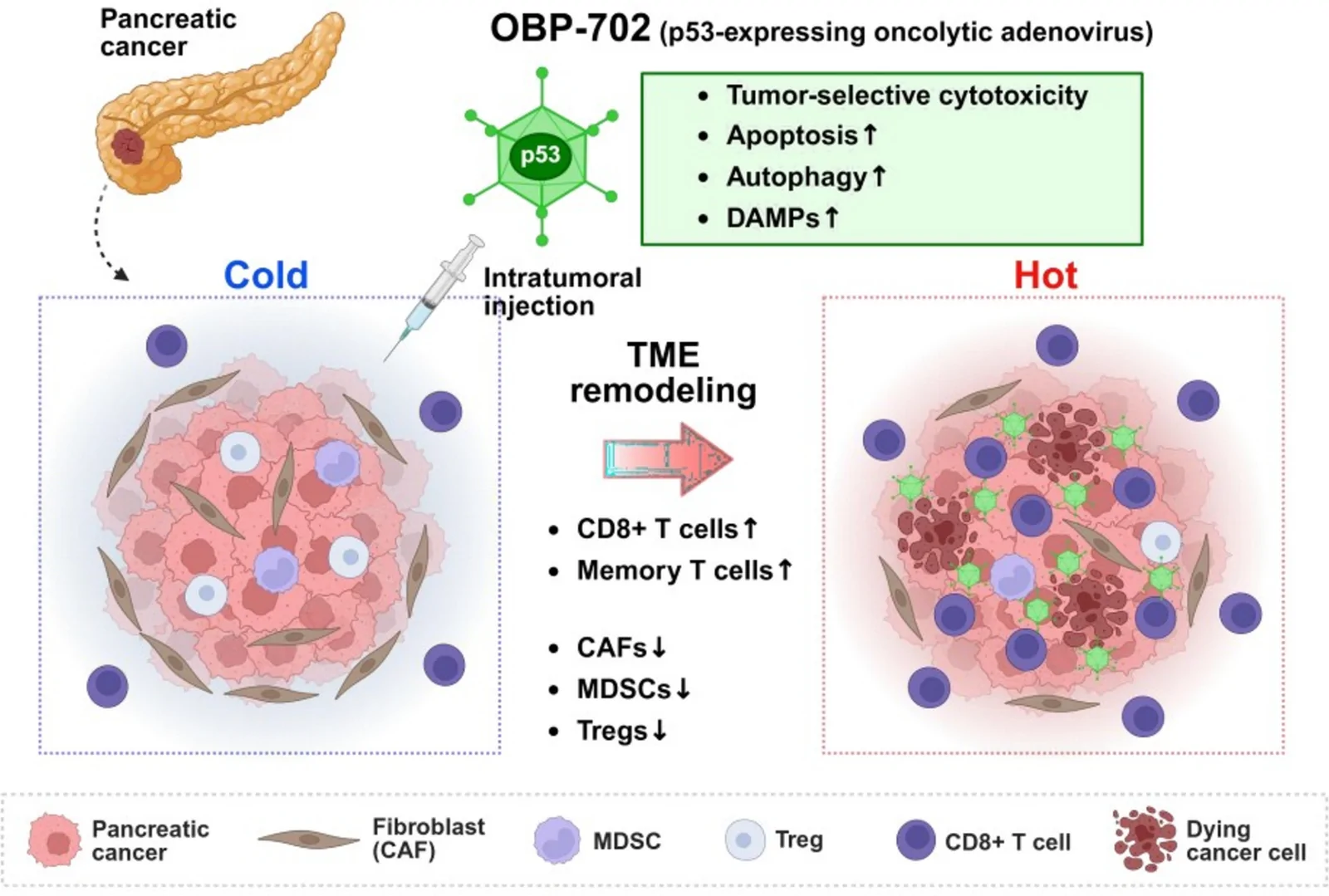
Schematic illustration of the therapeutic potential of OBP-702 in pancreatic cancer

This review outlined the global development status of oncolytic viruses for pancreatic cancer, as well as the preclinical research findings on OBP-702, a p53-armed oncolytic adenovirus we are currently developing (Fig. 2). Given the limited efficacy of ICIs in pancreatic cancer, despite their remarkable success in many other malignancies, oncolytic virotherapy is expected to serve as a potential breakthrough in this challenging cancer type. OBP-702, in particular, holds promise as a leading candidate in this emerging therapeutic field.
